# Effects of low‐to‐moderate alcohol supplementation on urinary estrogen metabolites in postmenopausal women in a controlled feeding study

**DOI:** 10.1002/cam4.1153

**Published:** 2017-09-06

**Authors:** Somdat Mahabir, Ruth Pfeiffer, Xia Xu, David J. Baer, Philip R. Taylor

**Affiliations:** ^1^ Environmental Epidemiology Branch Epidemiology and Genomics Research Program Division of Cancer Control and Population Sciences National Cancer Institute (NCI) Rockville Maryland; ^2^ Genetic Epidemiology Branch Division of Cancer Epidemiology and Genetics NCI Rockville Maryland; ^3^ Biostatistics Branch Division of Cancer Epidemiology and Genetics NCI Rockville Maryland; ^4^ Cancer Research Technology Program Leidos Biomedical Research, Inc. Frederick National Laboratory for Cancer Research Frederick Maryland; ^5^ Agricultural Research Service United States Department of Agriculture Beltsville Maryland

**Keywords:** Low‐to‐moderate alcohol, urinary estrogen metabolites, postmenopausal women, controlled feeding study

## Abstract

Heavy alcohol drinking is associated with increased breast cancer risk, but associations with low‐to‐moderate alcohol consumption are less clear and the biological mechanisms are not well defined. The objective of this study was to evaluate the effects of 8 weeks of low (15 g/d) and moderate (30 g/d) alcohol ingestion on concentrations of 15 urinary estrogen metabolites (EMs) in postmenopausal women (*n* = 51) in a controlled feeding study with a randomized crossover design. Compared to no alcohol, 15 g/day for 8 weeks had no effect on urinary EMs. However, compared to no alcohol, 30 g/day for 8 weeks decreased urinary 2‐hydroestrone (2‐OHE1) by 3.3% (*P *=* *0.055) and increased 16‐epiestriol (16‐EpiE3) by 26.6% (*P *=* *0.037). Trends for reduced urinary 2‐OHE1 (*P *=* *0.045), reduced ratio of 2‐OH:16OH pathways (*P *=* *0.008), and increased 16‐EpiE3 (*P *=* *0.035) were observed as alcohol ingestion increased from 0 g to 15 g to 30 g/d. Moderate alcohol consumption for 8 weeks had modest effects on urinary concentrations of 2‐OHE1 and 16‐EpiE3 among postmenopausal women in a carefully controlled feeding study.

## Introduction

The link between low‐to‐moderate alcohol consumption and postmenopausal breast cancer risk remains unclear, possibly because of limitations in epidemiological study designs. Since exposure to endogenous and exogenous estrogens and increased urinary estrogens 1 are important risk factors for breast cancer, clear evidence from controlled trials that low‐to‐moderate alcohol consumption increases concentrations of estrogens related to breast cancer would suggest a mechanism by which alcohol dose and duration of intake could increase risk, and provide support for a causal relationship.

Metabolism of parent estrogens, estrone (E1) and estradiol (E2) leads to the production of several estrogen metabolities (EM) in the urine. Endogenous estrogen metabolism pathways have previously been described 2, 3. Several of the estrogen metabolites in urine are hypothesized to increase breast cancer risk due to their estrogenic and genotoxic activities 3, 4. Mechanistically, estrogens may increase breast cancer risk in postmenopausal women via DNA damage 5, regulation of cell signaling pathways 6, and regulation of angiogenesis 7, possibly by an estrogen‐driven angiogenic switch 8.

From the Women's Alcohol Study (WAS), we previously reported increased serum estrone sulfate and DHEAS concentrations after 8 weeks 9 and as short as 4 weeks 10 of low (15 g/d) to moderate (30 g/d) alcohol consumption. We now report on the effects of low‐to‐moderate alcohol supplementation for 8 weeks on the concentrations of 15 urinary estrogen metabolites (EMs) including 2, 4, and 16‐hydroxylation pathways. Our secondary objective was to assess the effects of obesity on concentrations of urinary estrogen metabolites within each alcohol treatment group because there is considerable evidence that the risk of postmenopausal breast cancer is independently increased by obesity 11.

## Subjects and Methods

### The Women's Alcohol Study

Details of the postmenopausal WAS has previously been described 9. In brief, the WAS feeding study used a crossover design in which 65 healthy postmenopausal women were randomly assigned to control (0 g), 15 g/d and 30 g/d alcohol treatments in random order with a 2–5 weeks no alcohol washout periods between treatments. For the 0‐ and 15‐g alcohol periods, energy from alcohol was replaced with carbohydrates (Polycose and soft drinks). Of the 65 women, 63 began the controlled feeding study and 53 successfully completed it. Blood and urine samples were collected at baseline and week 8 of the study and were stored at −85°C. At the end of each 8‐week period, three 24‐h urine samples were collected, volume was measured, and aliquots were pooled for analysis.

### Assessment of urinary estrogen metabolites

At the end of each 8‐week period, three 24‐h urine samples were collected and aliquots were pooled and stored at −85°C until thawed for analysis. Samples were sent in 10 batches to the Laboratory of Proteomics and Analytical Chemistry, Leidos Biomedical Research, Inc. for measurement using a high‐performance liquid chromatography‐tandem mass spectrometry (LC‐MS/MS) method which measures EMs 12. Urinary EM concentrations were adjusted for creatinine.

For the LC–MS/MS assays, four blinded quality control (QC) samples were inserted in each of the 10 batches of urine samples analyzed. The coefficients of variation (CVs) within batches were as follows: 16α‐Hydroxyestrone (16α‐OHE1) (0.06–2.4%), 16‐Epiestriol (16‐epiE3) (0.09–2.1%), 16‐Ketoestradiol (16‐ketoE2) (0.03–1.7%), 17‐Epiestriol (17‐epiE3) (0.14–2.5%), 2‐Methoxyestrone (2‐MeOE1) (0.04–1.4%), 2‐Methoxyestradiol (2‐MeOE2) (0.3–4.6%), 2‐Hydroxyestrone (2‐OHE1) (0.03–0.7%), 2‐Hydroxyestradiol (2‐OHE2) (0.2–4.0%), 2‐Hydroxyestrone‐3‐methyl ether (3‐MeOE1) (0.1–3.2%), 4‐Methoxyestrone (4‐MeOE1) (0.1–2.6%), 4‐Methoxyestradiol (4‐MeOE2) (0.3–6.7%), 4‐Hydroxyestrone (4‐OHE1) (0.3–1.2%), Estrone (E1) (0.01–1.5%), Estradiol (E2) (0.2–2.2%), and Estriol (E3) (0.05–1.3%).

### Statistical analyses

Urinary EM concentrations were transformed using the Box‐Cox transformation to normalize their distributions. Mixed models were fitted to the transformed EMs adjusted for race and age in four categories defined by quartiles (≤53.9, 54–58.2, 58.3–63.6, >63.6 years), body mass index (BMI), and baseline values for each analyte using restricted maximum likelihood. In analyses that assessed the impact of BMI on EM concentrations, we fit separate linear regression models to each alcohol category adjusted for age and race, if appropriate. For the trend test, BMI categories were coded as 0, 1, and 2, and a Wald *P*‐value was calculated. Statistical analyses were conducted using SAS software (SAS/Stat version 9.1; SAS Institute, Cary, NC, USA).

## Results

Fifty‐one women had urinary EM measurements for all three treatments, as well as baseline urinary EM. Characteristics of the 51 study participants and their baseline urinary EM measurements are shown in Table 1. At baseline, as expected, there was wide variation in urinary EM concentrations overall and in Whites and Blacks.

**Table 1:** Characteristics and urinary estrogen metabolite (EM) levels of participants at baseline (*n* = 51 subjects)

Characteristic	Median 19
Age (y)	58.1 (49.2–78.8)
Height (cm)	163.1 (152.1–179.7)
Weight (kg)	73.2 (42.1–117.4)
BMI (kg/m^2^)	26.9 (17.8–42.5)
Years since last menses	12.0 (0.0–38.0)
Parity, no. of live births	2.0 (0.0–8.0)
	Number (%)
Educational level	
<High school	5 (10.0)
High school graduate	17 (34.0)
College/graduate work	28 (56.0)
Parous	
Yes	43 (84.3)
No	8 (15.7)
Menopause type	
Natural	42 (82.3)
Hysterectomy	9 (17.6)
Urinary EM (pmol/mg creatinine)	Median 19
Total parent estrogens and metabolites	36.30 (7.26–198.63)
Parent estrogens	7.46 (0.61–68.57)
Estrone (E1)	6.01 (0.31–56.21)
Estradiol (E2)	1.50 (0.31–12.36)
2‐Hydroxylation pathway	
2‐Hydroxylation pathway catechols	9.24 (1.70–63.41)
2‐Hydroxyestrone (2‐OHE1)	7.41 (1.48–53.16)
2‐Hydroxyestradiol (2‐OHE2)	5.89 (0.51–45.70)
2‐Hyroxylation pathway methylated catechols	1.25 (0.12–7.47)
2‐Methoxyestrone (2‐MeOE1)	1.74 (0.21–10.24)
2‐Methoxyestradiol (2‐MeOE2)	1.23 (0.05–8.47)
2‐Hydroxyestrone‐3‐methyl ether (3‐MeOE1)	0.19 (0.03–0.97)
	0.27 (0.01–0.96)
4‐Hydroxylation pathway	
4‐Hydroxylation pathway catechols	1.06 (0.11–17.77)
4‐Hydroxyestrone (4‐OHE1)	a
4‐Hydroxylation pathway methylated catechols	0.90 (0.01–16.99)
4‐Methoxyestrone (4‐MeOE1)	0.19 (0.03–1.86)
4‐Methoxyestradiol (4‐MeOE2)	0.14 (0.002–1.85)
	0.03 (0.002–0.22)
16α‐Hydroxylation pathway	
16α‐Hydroxyestrone (16α‐OHE1)	16.87 (4.34–73.35)
Estriol (E3)	3.38 (0.80–15.30)
17‐Epiestriol (17‐epiE3)	6.91 (0.49–38.10)
16‐Ketoestradiol (16‐ketoE2)	0.37 (0.02–2.01)
16‐Epiestriol (16‐epiE3)	4.62 (1.02–34.41)
	1.21 (0.13–7.02)

a 4‐Hydroxyestrone is the only 4‐Hydroxylation pathway catechol we measured; therefore, the medians and ranges are the same for both these measures.

Table 2 shows participants’ mean urinary EM concentrations and mean differences after consumption of no alcohol (placebo), 15 g/d, and 30 g/d alcohol. Low (15 g/d) alcohol consumption for 8 weeks versus the placebo had no effect on total parent estrogen metabolites or any of the 15 urinary EM concentrations. However, moderate (30 g/d) alcohol consumption (compared to none) decreased 2‐OHE1 by 3.3% (*P *=* *0.055) and increased 16‐epiE3 by 26.6%. (*P *=* *0.037). Trends for reduced urinary 2‐OHE1 (*P *=* *0.0454), a reduced ratio of the 2‐OH:16‐OH pathways and increased 16‐epiE3 (*P *=* *0.0347) were observed as alcohol dose increased from 0 g to 15 g to 30 g/d. There were no differences in the findings by race.

**Table 2:** Alcohol effects: mean urinary estrogen metabolites (EM) concentrations (pmol/mg creatinine), mean differences (delta), and 95% CI in urinary EM between no alcohol and when consuming 15‐ and 30 g/day

	No alcohol mean (95% CI)	0 vs. 15 g/day delta (95% CI)	0 vs. 30 g delta (95% CI)	*P*‐trend
Week 8				
Total parent estrogens and metabolites	45.50 (36.96–54.05)	0.74 (−4.41–5.90)	2.80 (−3.83–9.44)	0.75
Parent estrogens	10.45 (8.51–12.40)	0.23 (−0.50–0.98)	0.69 (−0.65–2.03)	0.74
Estrone (E1)	7.92 (6.42–9.42)	0.17 (−0.44–0.79)	0.44 (−0.57–1.45)	0.58
Estradiol (E2)	2.54 (2.03–3.04)	0.06 (−0.18–0.31)	0.25 (−0.12–0.62)	0.13
2‐Hydroxylation pathway	13.67 (11.75–15.59)	0.18 (−1.52–1.89)	−0.05 (−2.57–2.47)	0.41
2‐Hydroxylation pathway catechols	11.27 (9.44–13.11)	0.26 (−1.37–1.90)	−0.17 (−2.28–1.93)	0.26
2‐Hydroxyestrone (2‐OHE1)	9.07 (7.19–10.94)	0.19 (−1.37–1.74)	−0.30 (−2.11–1.50)^a^	0.045
2‐Hydroxyestradiol (2‐OHE2)	2.21 (1.72–2.70)	0.08 (−0.23–0.39)	0.13 (−0.33–0.59)	0.67
2‐Hydroxylation pathway methylated catechols	2.40 (1.96–2.83)	−0.07 (−0.35–0.19)	0.11 (−0.36–0.60)	0.95
2‐Methoxyestrone (2‐MeOE1)	1.72 (1.37–2.06)	−0.01 (−0.20–0.17)	0.13 (−0.23–0.49)	0.99
2‐Methoxyestradiol (2‐MeOE2)	0.28 (0.22–0.34)	0.01 (−0.04–0.06)	0.02 (−0.05–0.08)	0.78
2‐Hydroxyestrone‐3‐methyl ether (3‐MeOE1)	0.40 (0.23–0.57)	−0.07 (−0.24–0.10)	−0.02 (−0.21–0.16)	0.87
4‐Hydroxylation pathway	4.93 (−0.90–10.78)	−2.05 (−8.01–3.89)	−0.15 (−2.80–2.48)	0.27
4‐Hydroxylation pathway EM catechols	b	b	b	b
4‐Hydroxyestrone (4‐OHE1)	4.67 (−1.12–10.47)	−2.03 (−7.99–3.92)	−0.16 (−2.80–2.47)	0.78
4‐Hydroxylation pathway methylated catechols	0.26 (0.20–0.32)	−0.02 (−0.06–0.01)	0.004 (−0.03–0.04)	0.20
4‐Methoxyestrone (4‐MeOE1)	0.20 (0.14–0.26)	−0.02 (−0.05–0.02)	−0.001(−0.03–0.03)	0.71
4‐Methoxyestradiol (4‐MeOE2)	0.06 (0.05–0.07)	−0.01 (−0.02–0.01)^a^	0.01 (−0.01–0.02)	0.96
16α‐Hydroxylation pathway	16.43 (14.50–18.35)	2.37 (0.32–4.42)	2.32 (−0.14–4.80)	0.14
16α‐Hydroxyestrone (16α‐OHE1)	3.31 (2.86–3.76)	0.07 (−0.19–0.34)	0.10 (−0.29–0.49)	0.92
Estriol (E3)	7.39 (6.27–8.51)	0.44 (−1.64–2.53)	1.39 (−0.02–2.80)	0.15
17‐Epiestriol (17‐epiE3)	0.41 (0.28–0.54)	−0.001 (−0.07–0.07)	−0.02 (−0.13–0.08)	0.48
16‐Ketoestradiol (16‐ketoE2)	4.07 (3.54–4.61)	0.36 (0.003–0.71)	0.53 (−0.08–1.14)	0.25
16‐Epiestriol (16‐epiE3)	1.24 (1.07–1.41)	0.01 (−0.03–0.23)	0.33 (−0.14–0.68)^c^	0.035
Ratio of 2‐OH:16‐OH pathways	0.91 (0.77–1.05)	−0.07 (−0.18–0.03)	0.10 (−0.20–−0.00007)	0.008

Data shown in the table are crude (unadjusted) arithmetic means and differences. *P*‐trends are from mixed linear models adjusted for age, race, and BMI.

a *P* = 0.055 (from *t*‐test based on log‐transformed data).

b 4‐Hydroxyestrone is the only 4‐Hydroxylation pathway catechol we measured; therefore, results are the same for both of these measures.

c *P* = 0.037 (from *t*‐test based on log‐transformed data).

Compared to normal weight women (BMI ≤ 25; *n* = 15), overweight (BMI > 25 to ≤30; *n* = 20) and obese (BMI > 30; *n* = 15) women both showed a consistent trend (*P *<* *0.05) for increased urinary excretion of E1 and E2. Overweight and obese women also trended to higher urinary excretion of E3 and 16‐epiE3 in the no alcohol group (data not shown).

## Discussion

This is the first study to use a randomized, controlled, crossover trial to determine the effects of low‐to‐moderate alcohol ingestion on urinary EMs. Metabolism of the parent estrogens, E1 and E2 leads to the production of estrogen metabolities which can be measured in the urine, and several of these urinary metabolites are hypothesized to influence breast cancer risk due to their estrogenic, mitogenic, and genotoxic activities.

In this study, we found that moderate alcohol consumption had a modest but discernable influence on estrogen metabolism, as evidenced by decreased urinary concentrations of 2‐OHE1 and increased 16‐epiE3. Low‐to‐moderate alcohol doses did not affect any of the other 13 urinary EMs measured in the study. Our results also indicate that the alcohol effect on EM appeared to differ by pathway, apparently stimulating the 16α‐hydroxylation pathway and leading to an increase in the end‐product 16‐epiE3, while suppressing the 2‐hydroxylation pathway resulting in a decrease in 2‐OHE1 (Table 2). None of the other pathways or individual EMs were affected by low‐to‐moderate alcohol ingestion.

Since we measured EM in urine samples (excretion), our data are not directly comparable to estrogens in serum samples (circulation). We previously reported from the WAS that low‐to‐moderate alcohol consumption increased serum concentrations of estrone sulfate and DHEAS 9, 10. There is also evidence that serum EMs increase postmenopausal breast cancer risk 13. Recent evidence indicates that postmenopausal breast cancer risk increases with higher levels of 16‐epiE3 and decreases with lower levels of 2‐OHE1 in different prospective cohorts 4. Since moderate alcohol consumption in our study of postmenopausal women increased 16‐epiE3 and lowered 2‐OHE1, these results imply that moderate drinking may have dual effects on later breast cancer development, but this finding needs confirmation. We also assessed alcohol effects on the ratio of the 2‐OH:16‐OH pathways, a common metric in previous breast cancer epidemiology research 4, 14, and found that the ratio of these pathways decreased monotonically as alcohol dose increased from 0 g to 15 g to 30 g/d. To date, only one prospective study of urine concentrations of the ratio of the 2‐OH:16‐OH pathways in postmenopausal breast cancer risk has been published and no association was reported 14. Since even low‐dose alcohol consumption increases breast cancer risk 15, the effects of low‐to‐moderate drinking on breast cancer risk probably have biological effects other than on EM. There is also strong evidence that low‐to‐moderate levels of alcohol consumption protect against heart disease 16, so it is important to determine both the harms and benefits of light drinking.

We also assessed the effects of BMI on urinary estrogen metabolites measured in the study and found a consistent trend for increased concentrations of E1 and E2 with overweight and obesity within all alcohol doses. The main source of estrogen in postmenopausal women is from the conversion of androgens in the adipose tissue, and this presumably leads to higher exposure of breast cells to estrogens. Therefore, postmenopausal women with higher BMI are expected to have higher concentrations of estrogens. We and others 17, 18 have previously reported that postmenopausal women with higher levels of adiposity have higher levels of serum estrogen concentrations.

There are obvious strengths and limitations to the current research. Unlike observational studies in which alcohol dose and type are estimated based on questionnaires, we provided precise doses for a known duration under controlled diet conditions of weight maintenance. Controlled dietary conditions are important because diet has been shown to influence hormone concentrations, diet is highly variable in Western society, and our methods for measuring dietary intake in free‐living (and free eating) persons (typically by self‐reports via questionnaires) are poor. In addition, the crossover design of the study allowed participants to serve as their own controls and to increase power. However, this is a small study and the results may be affected by multiple comparisons.

In conclusion, our results suggest that moderate alcohol consumption, equivalent to two drinks per day, for 8 weeks influenced EM. We observed increased urinary 16‐epiE3 and decreased 2‐OHE1, suggesting that alcohol may affect EM pathways differently, stimulating some and suppressing others.

## Conflict of Interest

None declared.
